# Dynamics on higher-order networks: a review

**DOI:** 10.1098/rsif.2022.0043

**Published:** 2022-03-23

**Authors:** Soumen Majhi, Matjaž Perc, Dibakar Ghosh

**Affiliations:** ^1^ Department of Mathematics, Bar-Ilan University, Ramat-Gan 5290002, Israel; ^2^ Faculty of Natural Sciences and Mathematics, University of Maribor, Koroška cesta 160, 2000 Maribor, Slovenia; ^3^ Department of Medical Research, China Medical University Hospital, China Medical University, Taichung, Taiwan; ^4^ Complexity Science Hub Vienna, Josefstödter Straße 39, 1080 Vienna, Austria; ^5^ Alma Mater Europaea, Slovenska ulica 17, 2000 Maribor, Slovenia; ^6^ Physics and Applied Mathematics Unit, Indian Statistical Institute, 203 B. T. Road, Kolkata 700108, India

**Keywords:** higher-order networks, synchronization, cooperation, dynamics

## Abstract

Network science has evolved into an indispensable platform for studying complex systems. But recent research has identified limits of classical networks, where links connect pairs of nodes, to comprehensively describe group interactions. Higher-order networks, where a link can connect more than two nodes, have therefore emerged as a new frontier in network science. Since group interactions are common in social, biological and technological systems, higher-order networks have recently led to important new discoveries across many fields of research. Here, we review these works, focusing in particular on the novel aspects of the dynamics that emerges on higher-order networks. We cover a variety of dynamical processes that have thus far been studied, including different synchronization phenomena, contagion processes, the evolution of cooperation and consensus formation. We also outline open challenges and promising directions for future research.

## 1. Introduction

The theory of complex networks [1,2] provides us with a framework for investigating the structure and dynamics of interacting systems. Indeed, network science has proven highly efficient in elucidating the dynamics of complex systems arising from many different contexts in the physical, biological as well as technological and social sciences [3,4]. Many key developments have been made in view of identifying and improving the concepts of association among the constituents of a network. To illustrate, the necessity of considering the links of networks that are different in nature has led to the formulation and detailed analysis of multilayer networks [5]. Further, time-varying networks [6,7] are investigated in which interactions do not persist for all the course of time, rather they arise or vanish over time. It is unquestionably true that all these developments have helped us to perceive many scenarios better, but we have specifically assumed dyadic or pairwise interactions as the backbone for connections among the units of the system. However, for a complete explanation of many complex systems, one needs to further improve the structural modelling of networked systems [8,9]. For instance, group interactions take place predominantly in systems arising in neurobiology [10,11], social systems [9,12] and ecology [13,14]. The network framework has been intrinsically limited to explanation through pairwise interactions, which are sufficient only to model the dyadic relationships, and so larger group interactions need a better formulation for networked systems [15–17]. It has been argued that higher-order structures, namely hypernetworks and simplicial complexes, are excellent frameworks to characterize the organization of many social, biological and other scenarios encoded in group interactions of three or more constituents [8,18].

Previously, not much attention has been paid to the analysis of networks exposed to higher-order interactions. However, a significant number of recent advances have demonstrated that the incorporation of higher-order architecture can remarkably improve our understanding and prediction ability of their dynamics. The studies related to these higher-order structures have thus come to the forefront of network dynamics research. Among some highly significant studies on higher-order networks, the one by Benson *et al.* [19], which investigates datasets from different disciplines ranging from various social networks to biology and demonstrates a variety of characteristics of the higher-order structures emerging therein, is particularly noteworthy. In addition, the problem of higher-order link prediction is formulated as this has been found to be essentially different from the traditional dyadic link prediction [20]. This issue of link prediction in networks subject to the presence of higher-order structures is also studied in [21] while dealing with different link prediction algorithms. The inverse problem of inferring higher-order interactions from observational data has also been discussed in [22], whereas higher-order interactions are inferred from the traditional dyadic interaction network data through a Bayesian approach in [23]. An analytical treatment (statistically validated hypergraphs) is propounded [24] for the problem of finding the most important relationships among the constituents of a higher-order network. Tie strengths are quantified by considering higher-order interactions encoded by groups of three or more individuals in social networks by the measure ‘Edge PageRank’ [25]. This measure has proved to be much more efficacious than the traditional approaches for detection of tie strength. A vector centrality measure is proposed for higher-order networks with the aim of identifying the most influential nodes in the system [26].

Different models of higher-order networks [27] have been developed so far. Detailed analysis of models of growing simplicial complexes [28–30] is presented, built upon the concept of ‘network geometry with flavour' (NGF) [31,32]. The models yield exponential or scale-free generalized degree distribution based on the non-preferential or preferential attachment rules. A ‘simplicial activity-driven model’ [33] is proposed and analysed that captures both the higher-order structure and the temporal nature of interactions among the nodes. A ‘simplicial configuration model’ [34,35] with a uniform Markov chain Monte Carlo sampler is introduced, even for arbitrary degree and size distributions [36]. In order to provide formalism for modelling heterogeneous, polyadic network data, the configuration models of random hypernetworks [37] and the annotated hypergraph model [38] are presented as a generalization of directed graphs. On the other hand, higher-order network set-ups are used to generalize the formalism of structural controllability to time-varying networks [39], for which both synthetic and real-world datasets are examined to illustrate the minimum time required to control the concerned systems. Group research collaborations of three or more individuals are illustrated through a higher-order interaction framework and further encoded under multilayer formalism with collaboration data taken from different research disciplines [40]. Further, heterogeneous dynamics of higher-order structures in time-varying social networks is examined for a number of social datasets [41].

Because of the perception that higher-order clusters are particularly important, the concept of higher-order clustering coefficients is introduced in [42], which quantifies the closure probability of higher-order cliques. This measure is used to examine the clustering behaviour of both model and real-world networks. The problem of clustering in hypernetworks with categorical edge labels can be addressed with a procedure based upon the combinatorial objective function [43]. The efficiency of this mechanism is validated for edge-label community detection and clustering with time-stamped data. Simplicial communities are detected from real-world data of social networks while showing that the spectra of the Hodge Laplacian encodes the communities [44]. A stochastic generative model is introduced to hypernetwork clustering with heterogeneous node degree and hyperlink size distribution [45]; this is shown to be highly scalable and efficient with the utilization of synthetic and various real-world data. Tudisco & Higham [46] have recently come up with their study of a family of spectral centrality measures in order to recognize important nodes and hyperlinks in hypernetworks, which extends the existing concepts for dyadic interactions. However, the formalism constructed by Veldt *et al.* [47], using hypernetworks to measure ‘homophily’, unravels that homophilous group configurations are impossible owing to the combinatorial impossibility of hypernetworks.

Concerning dynamical processes, frameworks for hypernetwork robustness and analysis of higher-order percolation processes [48–50] are put forward for multiplex hypernetworks. Analogous to the largest eigenvalue of the matrix representing the interaction structure of a network built upon dyadic connections, the concept of an ‘expansion eigenvalue’ for hypernetwork dynamical processes is proposed and approximated through a mean-field approach [51]. Quite interestingly, in the case of random interactions in ecological communities, the presence of higher-order species interactions can certainly alter the traditional relationship between diversity and stability [52]. For instance, even though dyadic interactions cause sensitivity to the species addition, four-way interactions result in sensitivity to the removal of species. Also, the merger of the dyadic and higher-order interaction induces both upper and lower bounds on the number of species. Moreover, there exists evidence of higher-order interactions stabilizing the dynamics in ecological communities [14] where interaction between species is influenced by other species. In both open and closed ecological communities, higher-order interactions have noticeable impacts that stabilize the dynamics for competitive models. Stochastic models under higher-order interactions help further in the sustained coexistence of species (see also [13] and references therein).

In the next section (§2), we briefly recall the basic definition of relevant terminology in higher-order interactions. We then start discussing the phenomenon of synchronization emerging in higher-order networked systems (see §3). Then, in §4, we move on to explore various social dynamics evolving over higher-order structures. Specifically, we investigate the processes of contagion dynamics, consensus formation and evolutionary game dynamics in §§4.1, 4.2 and 4.3, respectively. Section 5 deals with the dynamics of random walk and diffusion. Finally, §6 offers a summary and discussion about potential research in the future.

## 2. Basic concepts

*Hyperlink*: Hyperlink is the fundamental backbone of a higher-order network, which instead of joining only two nodes (for the traditional networks of pairwise interactions) can connect any number of nodes.

*Hypernetwork*: Hypernetwork is a generalization of the notion of network, and is composed of hyperlinks. This implies that a hypernetwork H can be considered as the pair (*V*, *E*) in which *V* is the set of nodes and *E* (a subset of the power set *P*(*V*) of *V*) is the set of hyperlinks.

*Simplex*: A *d*-dimensional simplex (or a *d*-simplex) is simply a set of *d* + 1 fully interacting nodes. Essentially, a 0-simplex is a node, a 1-simplex is a link, a 2-simplex is a triangle, a 3-simplex is a tetrahedron and so on.

*Simplicial complex*: Similar to the networks as a collection of links, a simplicial complex comprises simplices. From the context of hypernetworks, a simplicial complex is a particular type of hypernetwork that accommodates each subset of all the hyperlinks. This means that a simplicial complex S is a hypernetwork that fulfils the criterion that, for each *e* ∈ *E* and $\forall e^{\prime }\subseteq e\;(e^{\prime }\neq \emptyset )$, one also has *e*′ ∈ *E*.

**Figure 1.**
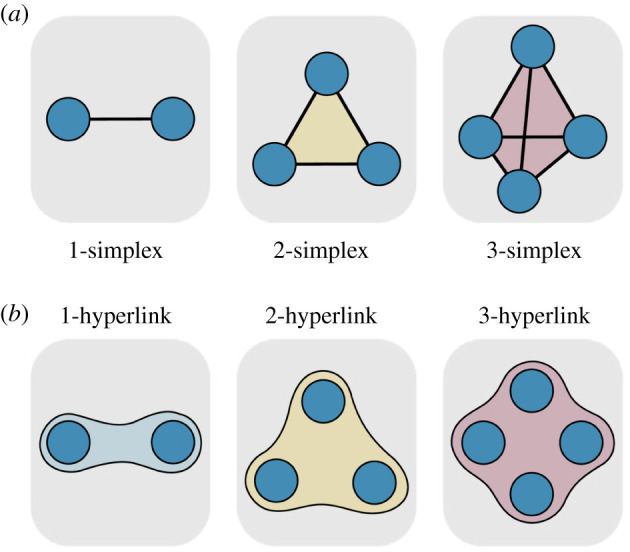
The higher-order building blocks, namely the simplices (*a*) and the hyperlinks (*b*) of dimension 1, 2 and 3. Adapted from [8].

In figure 1, examples of simplices (figure 1*a*) and hyperlinks (figure 1*b*) of dimension from 1 to 3 are depicted that clarify the higher-order building blocks upon which the higher-order networks are generally built.

In the next section, we focus on elaborating the principal findings and the novel effects in the dynamical processes that the higher-order interactions bring about, and hence can be of interest for the perception of a number of natural occurrences. We, however, mention here that we do not really distinguish between the dynamics on hypernetworks and the dynamics on simplicial complexes; rather, we present an excerpt of diverse dynamics on top of higher-order networks, in general.

## 3. Synchronization

The phenomenon of synchronization [53–58] corresponds to a process wherein interacting dynamical systems adjust certain properties of their motion to a common dynamics, and this interaction pattern plays decisive roles for the emergence of synchrony. Synchronization is considered to be one of the most important phenomena in complex dynamical network theory, having crucial applications in several physical, biological and technological systems. Hence, there has been a strong desire to explore different aspects of synchronization in coupled systems in the last two decades. However, only recently have the investigations tended towards higher-order interactions.

Specifically, three-body interactions in an ensemble of phase oscillators can give rise to an infinite number of multistable synchronized attractors beyond a critical interaction strength [59]. Simplicial complexes of large ensembles of interacting oscillators are considered while modelling the three-way interactions on top of a multilayer framework [60]. A continuum of abrupt transitions to desynchronization is observed therein as a result of multistability consisting of an infinite number of stable partially synchronized states. An analytical treatment is provided based upon dimensionality reduction through a variation of the Ott–Antonsen ansatz. In addition, synergistic effects of higher-order interactions of different order (namely, 1-, 2- and 3-simplex) on synchrony arising in heterogeneous Kuramoto phase oscillators is analysed [61]. Here, it has been shown that the interplay of these simplicial structures can yield abrupt transitions to both desynchronization and synchronization, and can stabilize strong synchrony even under repulsive pairwise interaction. The authors also enunciate the phenomena while dealing with UK power grid and macaque brain networks. Gambuzza *et al.* [62] have recently presented a general framework for the stability of synchronization in networks subject to higher-order interactions of any order. The authors demonstrate the existence of complete synchrony as an invariant solution and provide the necessary conditions for the synchronous solution. The generality of the proposed formalism is elucidated by considering a paradigmatic chaotic Rössler system and model systems pertinent to neurodynamics. Furthermore, cluster synchronization is studied in a model of a simplicial complex of Rössler oscillators.

Analysis of *D* ( ≥2)-dimensional Kuramoto dynamics on top of simplicial structures (1- and 2-simplices predominantly) is presented in [63]. Theoretical analysis and extensive numerical simulations are put forward wherein reasoning behind different synchronization patterns resulting from odd and even dimensions is explained [64]. Interestingly, discontinuous transition to desynchronization for any dimension at positive interaction strength, discontinuous transition for odd dimensions at zero coupling strength and the state of partial synchronization for all odd *D* (along with *D* = 2) at negative interaction strength are described. Further, a globally coupled ensemble of the *D*-dimensional Kuramoto oscillators consisting of only contrarians possesses collective synchrony in the absence of any conformists, if the underlying connection among the units goes beyond dyadic interactions [65]. This result, in particular, is forbidden in networks with only pairwise communications. A notably interesting formulation of the higher-order Kuramoto dynamics that designates interactions among oscillators placed not only on the nodes but also on the higher-dimensional simplices such as links, triangles etc. permits one to describe a topologically projected dynamics on lower- and higher-dimensional faces [66]. It has been shown that, besides a simple continuous transition to synchronization, with an adaptive coupling between the dynamics projected on the lower- and higher-dynamical phases the networked system exhibits *explosive* transition to synchrony.

Furthermore, the interaction between dynamical signals defined on nodes and links yields explosive topological synchronization wherein the phases ascribed on the nodes synchronize to the phases defined on the links at a discontinuous transition [67]. Detailed analytical treatment is provided that explores this scenario and the associated closed hysteresis loop in the limit of large size of the networks. Besides dealing with simplicial complexes, the model has been tested on the human and *Caenorhabditis elegans* connectomes. To be precise, a simplicial complex of *N*_[*n*]_ simplices of dimension *n* (i.e. *N*_[0]_ nodes, *N*_[1]_ number of links, *N*_[2]_ triangles, etc.), with ***B***_[*n*]_ as the *n*th incidence matrix for the *n*th boundary operator, are assumed. Then the phase vector $\theta =(\theta_{1},\theta_{2},\ldots ,\theta_{N_{\left[0\right]}})$ associated with the nodes obeys the following dynamical equation:

3.1 $$\dot{\theta }=\omega -\sigma B_{\left[1\right]}\sin(B_{\left[1\right]}^{T}\theta ),$$

where *σ* is the interaction strength, with each *ω*_*k*_ chosen from a given distribution, say a normal distribution $\omega_{i}∼N(\Omega_{0},1/\tau_{0})$. Consequently, the associated order parameter can be written as

3.2 $$R_{0}=\frac{1}{N_{\left[0\right]}}\left|\sum_{k=1}^{N_{\left[0\right]}}e^{i\theta_{k}}\right|.$$

The higher-order topological Kuramoto model defined on phases $\phi =(\phi_{l_{1}},\phi_{l_{2}},\ldots ,\phi_{l_{N_{\left[1\right]}}})$ associated with the links is described as

3.3 $$\dot{\phi }=\tilde{\omega }-\sigma B_{\left[1\right]}^{T}\sin(B_{\left[1\right]}\phi )-\sigma B_{\left[2\right]}\sin(B_{\left[2\right]}^{T}\phi ),$$

with ${\tilde{\omega }}_{l}∼N(\Omega_{1},1/\tau_{1})$ as the internal frequencies for the links. With the synchronization dynamics defined on the higher-order *n*( = 1)-dimensional signals, the projections ***ϕ***^[−]^ and ***ϕ***^[+]^ on the *n* − 1 simplices (i.e. nodes) and *n* − 2 simplices (i.e. triangles) are ***ϕ***^[−]^ = ***B***_[1]_***ϕ*** and $\phi^{\left[+\right]}=B_{\left[2\right]}^{T}\phi$, which, respectively, act according to the following dynamics:

3.4 $$\begin{array}{rl} {\dot{\phi }}^{\left[-\right]} & =B_{\left[1\right]}\tilde{\omega }-\sigma L_{\left[0\right]}\sin(\phi^{\left[-\right]})\\ \mathrm{and}\;\;{\dot{\phi }}^{\left[+\right]} & =B_{\left[2\right]}^{T}\tilde{\omega }-\sigma L_{\left[2\right]}^{\mathrm{down}}\sin(\phi^{\left[+\right]}), \end{array}\}$$

where $L_{\left[0\right]}=B_{\left[1\right]}B_{\left[1\right]}^{T}$ and $L_{\left[2\right]}^{\mathrm{down}}=B_{\left[2\right]}^{T}B_{\left[2\right]}$, and the corresponding order parameters obtain the forms:

3.5 $$\begin{array}{cc} & R_{1}^{\mathrm{down}}=\frac{1}{N_{\left[0\right]}}\left|\sum_{k=1}^{N_{\left[0\right]}}e^{i\phi_{k}^{\left[-\right]}}\right|\\ \mathrm{and}\; & R_{1}^{\mathrm{up}}=\frac{1}{N_{\left[2\right]}}\left|\sum_{k=1}^{N_{\left[2\right]}}e^{i\phi_{k}^{\left[+\right]}}\right|. \end{array}\}$$

Then, unlike the adaptive coupling between these two dynamics of the same dimension as in [66], here signals of different dimensions are coupled through the order parameters of the node and link dynamics (i.e. equations (3.2) and (3.5)). In particular, two models named nodes–links (NL) and nodes–links–triangles (NLT) are considered. The former is described as

3.6 $$\begin{array}{rl} \dot{\theta } & =\omega -\sigma R_{1}^{\mathrm{down}}B_{\left[1\right]}\sin(B_{\left[1\right]}^{T}\theta )\\ \mathrm{and}\;\dot{\phi } & =\tilde{\omega }-\sigma R_{0}B_{\left[1\right]}^{T}\sin(B_{\left[1\right]}\phi )-\sigma B_{\left[2\right]}\sin(B_{\left[2\right]}^{T}\phi ), \end{array}\}$$

with the projected dynamics following:

3.7 $$\begin{array}{rl} {\dot{\phi }}^{\left[-\right]} & =B_{\left[1\right]}\tilde{\omega }-\sigma R_{0}L_{\left[0\right]}\sin(\phi^{\left[-\right]})\\ \mathrm{and}\;\;{\dot{\phi }}^{\left[+\right]} & =B_{\left[2\right]}^{T}\tilde{\omega }-\sigma L_{\left[2\right]}^{\mathrm{down}}\sin(\phi^{\left[+\right]}). \end{array}\}$$

The latter is defined as

3.8 $$\begin{array}{rl} \dot{\theta } & =\omega -\sigma R_{1}^{\mathrm{down}}B_{\left[1\right]}\sin(B_{\left[1\right]}^{T}\theta )\\ \mathrm{and}\;\;\dot{\phi } & =\tilde{\omega }-\sigma R_{0}R_{1}^{\mathrm{up}}B_{\left[1\right]}^{T}\sin(B_{\left[1\right]}\phi )-\sigma R_{1}^{\mathrm{down}}B_{\left[2\right]}\sin(B_{\left[2\right]}^{T}\phi ), \end{array}\}$$

where the projected dynamics obeys

3.9 $$\begin{array}{rl} {\dot{\phi }}^{\left[-\right]} & =B_{\left[1\right]}\tilde{\omega }-\sigma R_{0}R_{1}^{\mathrm{up}}L_{\left[0\right]}\sin(\phi^{\left[-\right]})\\ \mathrm{and}\;\;{\dot{\phi }}^{\left[+\right]} & =B_{\left[2\right]}^{T}\tilde{\omega }-\sigma R_{1}^{\mathrm{down}}L_{\left[2\right]}^{\mathrm{down}}\sin(\phi^{\left[+\right]}). \end{array}\}$$

**Figure 2.**
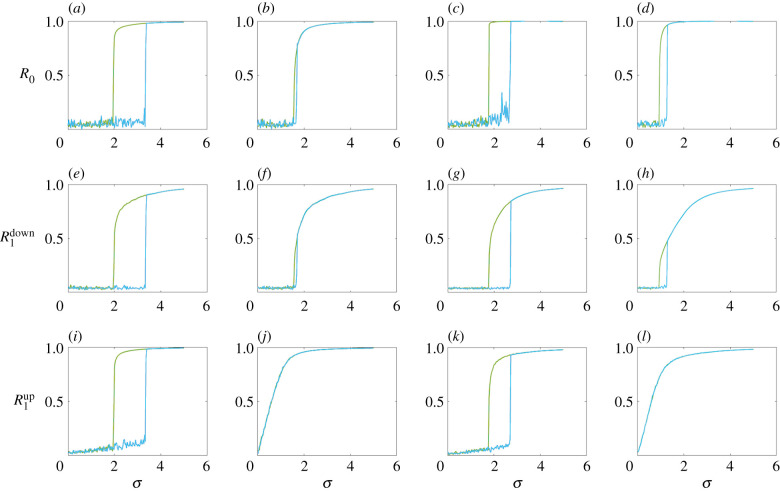
The evolution of the order parameters *R*_0_, $R_{1}^{\mathrm{down}}$ and $R_{1}^{\mathrm{up}}$ with respect to the coupling strength *σ*. The first two columns (from the left) correspond to the NGF model, with the last two columns representing the configuration model. Also, the second and fourth columns (from the left) represent the NL model whereas the first and third columns (from the left) correspond to the NLT model. The cyan lines designate the forward transitions and the green lines indicate the backward transitions. For both network models, *N*_[0]_ = 500. Further, $\Omega_{0}=\Omega_{1}=2$ and *τ*_0_ = *τ*_1_ = 1 are chosen. Adapted from [67].

Now, with these two dynamical models NL and NLT in hand, extensive numerical simulations are carried out on two models of simplicial complexes, namely the configuration model [35] and the NGF model [31]. Figure 2 displays the values of the order parameters *R*_0_, $R_{1}^{\mathrm{down}}$ and $R_{1}^{\mathrm{up}}$ as functions of the coupling strength *σ* in the top, middle and bottom panels, respectively. The first two columns correspond to the NGF model for flavour *s* = −1 and *d* = 3-dimensional simplicial complexes with an underlying power-law network (exponent *γ* = 3), whereas the last two columns are for the configuration model for the power-law (exponent *γ* = 2.8) generalized degree distribution, with *N*_[0]_ = 500 nodes for both cases. Moreover, the first and third (second and fourth) columns depict the outcomes for the NLT (NL) model. As confirmed, in both the scenarios, explosive transitions to the state of synchronization take place. The transitions occur along with the hysteresis loop formed by the forward and backward transitions. For the NLT model, all the order parameters *R*_0_, $R_{1}^{\mathrm{up}}$ and $R_{1}^{\mathrm{down}}$ show discontinuous transitions to synchrony at the same coupling strength. But in the case of the NL model, although *R*_0_ and $R_{1}^{\mathrm{down}}$ demonstrate discontinuous transitions for some critical interaction strength, $R_{1}^{\mathrm{up}}$ ensures an independent transition at zero coupling strength for both network models. This is because, in the NL model, the adaptive interaction couples only the phases ***ϕ***^[−]^ and ***θ***, and not the phase ***ϕ***^[+]^. For further details of the analytical treatment alongside the numerical results, see [67].

Synchronization in an ensemble of Kuramoto phase oscillators subject to the interplay of interactions built upon 1-simplex (i.e. the links) and the 2-simplex (i.e. the triangles) faces of homogeneous four-dimensional simplicial complexes is reported in [68]. In the presence of only dyadic interactions, increasing positive coupling strength leads to a continuous transition to complete synchrony, whereas negative coupling results in a partial synchronization. It needs to be mentioned here that no synchrony is observed for negative coupling in scale-free networks. Moreover, introduction of the higher-order (2-simplex) coupling impedes the synchrony induced by the pairwise interaction, and causes the hysteresis loop with abrupt transition to desynchronization for negative pairwise coupling. Also, in a recent work [69], the authors assume an adaptive higher-order (triadic) interaction formalism relying on the Hebbian learning mechanism in networks of Kuramoto phase oscillators and showed that such a coupling can induce the first-order transition to desynchronization. The presented scenario is further explained through a detailed mean-field analysis. Partial loss of synchronization can also be witnessed in a generalized Sakaguchi–Kuramoto model formed through the inclusion of linear and nonlinear frustrations in the simplicial Kuramoto model [70] and weights on the simplices. A precise mathematical framework is presented in the article apart from the computational results for this frustrated model on links while dealing with measures such as the order parameter and Hodge decomposition.

The stability of synchronization in ensembles of oscillators subject to higher-order interactions of any order and built upon any complex underlying hypernetwork structure with arbitrary coupling functions can be analysed through a general formalism of a *multiorder Laplacian* [71]. Different network set-ups are investigated, including the one that deals with both attractive and repulsive interactions [72]. Datasets ranging from synthetic to empirical are studied under this framework. Higher-order interactions embedded in clique complexes can optimize the collective synchronization in the Kuramoto model subject to equitable increases in the strength of the higher-order connections relative to the pairwise interactions [73]. Synchronization dynamics and formation of Turing patterns in nonlinear chaotic systems are studied in higher-order networks while using the master stability function (MSF) framework and analysing an appropriate combinatorial Laplacian [74]. The processes are examined for general hypernetworks with a heterogeneous distribution of hyperlinks, and are not restricted to any specific form of the coupling function. Different synchronization patterns from cluster synchrony to chimeras are realized in generalized networks, including multilayer networks, hypernetworks and time-varying networks through the simultaneous block diagonalization (SBD) approach [75]. MSF formalism has also been generalized for hypernetworks in [76] to treat the linear stability of the phenomenon of synchronization, where the special class of Laplace-type interactions has also been examined. The dynamical systems known as coupled map lattices are extended to the scenario of higher-order interactions, namely to the concept of coupled hypergraph maps [77]. The process of cluster synchronization is investigated in such a system through the analysis of a hypernetwork Laplacian for different chaotic discrete dynamical systems. Very recently, in [78] the authors consider three-body interactions along with the dyadic couplings for an interacting Hindmarsh–Rose neuronal model while deriving the necessary conditions for the emergence of synchrony by means of linear stability analysis.

## 4. Social dynamics

Diverse social processes have always been a major area of research in complexity science. A wide list of scenarios ranging from opinion, cultural and language dynamics to crowd behaviour, hierarchy formation, human dynamics, evolution of cooperation and social spreading is considerably influenced by peer-to-peer interaction among individuals embedded in social networks. Such contagion effects direct researchers to explore the dynamics from a mathematical point of view. For this purpose, network science has emerged to play the most significant role. In the past decade, we have witnessed a golden age in the study of social dynamics over networks, from different perspectives. The research communities have long been interested in the interactions between individuals leading to diverse emerging behaviour. However, as noted in the reviews by Castellano *et al.* [79] and Malmgren *et al*. [80], there are significant aspects of real social contagion phenomena that need to be captured with much more sophisticated approaches than before, from the perspective of both dynamics on networks and dynamics of networks. In the following, we go through different genres of studies of social dynamics exposed to higher-order interactions.

### 4.1. Contagion processes

In order to take into account the group interactions of different sizes, Iacopini *et al.* [81] formulated a higher-order simplicial model of social contagion. The model incorporates both pairwise and higher-order contacts, and thus combines the essences of both simple and complex contagion processes. Simplicial structure leads to a discontinuous transition to the endemic state and bistability emerges in which endemic and healthy states coexist. The former scenario has been demonstrated analytically along with numerical demonstrations on random Erdös–Rényi model and empirical higher-order networks. This model is then extended to the framework of temporal networks [82], in which dyadic and higher-order interactions can be formed and destroyed temporally. Going through a microscopic Markov chain approach it has been shown that the same number of infectious seeds may or may not generate an endemic state, which actually depends on the temporal properties of the underlying network. The impact of degree heterogeneity on the simplicial contagion over time-varying higher-order networks is also investigated in the article thereafter. Social contagion dynamics is further investigated on hypergraphs in de Arruda *et al*. [83]. The authors particularly embodied the critical-mass dynamics into the previously framed model of Iacopini *et al*. [81]. Analytical and numerical results are presented to show the emergence of continuous and discontinuous transitions together with bistable regimes and hysteresis.

In addition to demonstrating that the standard network-generating algorithms with tunable clustering characteristics can yield diverse higher-order structures so that dynamics can differ on the networks with the same clustering and degree distribution profiles, Ritchie *et al*. [84] formulated a new metric for measuring order-4 structures. The authors emphasize that the higher-order structural differences (arising in networks possessing the same clustering) have consequences for epidemic prevalence and epidemic threshold while dealing with susceptible–infected–susceptible (SIS) and susceptible–infected–recovered (SIR) dynamical models. Landry & Restrepo [85] studied the dynamics of an SIS model on hypergraphs by means of hyperdegree-based mean-field analysis on networks with higher-order interactions. Both degree-correlated and -uncorrelated cases are analysed, and it is shown that the abrupt first-order transitions can be suppressed through heterogeneous degree distribution of the dyadic interactions under certain assumptions on degree correlations. Besides inferring the conditions for bistability and hysteresis, the issues related to higher-order healing and the ‘hipster effect’ are further discussed in their article.

Lately, a higher-order model has been developed that addresses a number of issues that have been mostly neglected in epidemic modelling. Heterogeneity that arises in environments such as offices and households and the temporal heterogeneity in participation of the individuals in these environments are analysed [86]. This heterogeneous exposure subject to a minimal infective dose yields a universal nonlinear relation between the risk of infection and the infected contacts, challenging the prevalent assumption of a linear relationship between these two. As a result, a discontinuous transition to an epidemic outbreak takes place and a bistable regime emerges as well in which outbreak and healthy states coexist.

**Figure 3.**
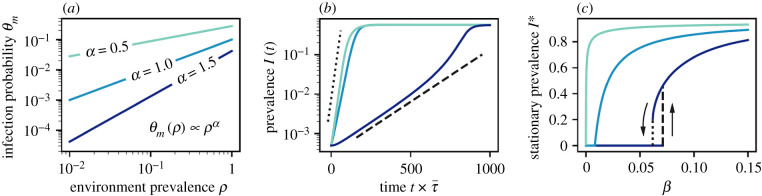
(*a*) Infection kernel with *β* = 0.1 where the infection probability *θ*_*m*_ has a power-law scaling *θ*_*m*_(*ρ*) ∝ *ρ*^*α*^. (*b*) Supralinear kernels *ν* > 1 result in a superexponential growth of the prevalence *I*(*t*). *β* = 0.0005, 0.025 and 0.077 for *ν* = 0.5, 1.0 and 1.5 are chosen, respectively, where $\bar{\tau }$ is the median exposure period. (*c*) Stationary prevalence I* with respect to *β*, where a continuous phase transition appears for sublinear and linear kernels *ν* ≤ 1 and a discontinuous phase transition arises with a bistable solution for supralinear kernels *ν* > 1. Poisson distributions are opted for both the distribution $\bar{P}(m)$ of the size *m* of the hyperlinks and the distribution $\tilde{P}(k)$ of the hyperdegree *k* of the individuals with 〈*m*〉 = 10 and 〈*k*〉 = 5, respectively, and *μ* = 0.05. Adapted from St-Onge *et al*. [86].

Let us consider the interaction framework to be a hypernetwork in which the environments are defined by hyperlinks of *m* individuals where each individual is incident to *k* hyperlinks. A discrete-time process (*t* = 1, 2, …) is then assumed where for each environment a participation time *τ* ∈ [1, *τ*_max_] is chosen for each individual. Then if a susceptible individual is participating in an *m*-sized environment for duration *τ* under the presence of a fraction *ρ* of the other infected participants, it receives an infective dose *l* ∈ [0, ∞) from the infected individuals, according to the distribution *f*(*l*; *λ*), where *λ* ≡ 〈*l*〉. Similar to the threshold models, it is then considered that someone develops the disease when *l* > *K* and *θ*_*m*_(*ρ*) is the infection kernel that represents the probability of getting the infection in an *m*-sized environment subject to a fraction *ρ* of other infected participants. Also, *w* ≤ *τ*_max_ is the clearing window which represents the characteristic time for the immune system to be free of any dose *l* < *K*. Then, for heterogeneous exposure periods described by a Pareto distribution *P*(*τ*) ∝ *τ*^−*α*−1^, where *α* > 0 and the characteristic time to be infected is *τ*_*c*_ ≡ *K*/*βg*(*m*)*ρ* (*g*(*m*) governs the number of contacts frequented by *m* nodes and *β* is a dose accumulation rate), *θ*_*m*_(*ρ*) takes the form $\theta_{m}(\rho )∼D_{\alpha }{\tau_{c}}^{-\alpha }\propto \rho^{\alpha }$ [86], with *D*_*α*_ being a constant. This is demonstrated in figure 3*a* for an exponential dose distribution *f*(*l*; *λ*) = e^−*l*/*λ*^, *g*(*m*) = 1 and *w* → ∞ for different values of *α*.

Further, the effects of nonlinear infection kernels are discussed while considering an SIS model with recovery rate *μ*. With a degree-based mean-field approximation, for the marginal probability *ρ*_*k*_(*t*) of an individual to be infected at time *t* and $\tilde{P}(k)$ as the distribution of hyperdegree *k* of the individuals, the global prevalence is $I(t)=\sum_{k}\rho_{k}(t)\tilde{P}(k)$. The temporal evolution of this prevalence is portrayed in figure 3*b*, which mirrors the impact of the nonlinear infection kernel. Specifically, superexponential growth is observed whenever *ν* > 1, (*θ*_*m*_(*ρ*) ∝ *ρ*^*ν*^) whereas the growth is regular exponential until saturation if *ν* ≤ 1. Finally, figure 3*c* depicts the stationary prevalence I* as a function of *β*. The absorbing state I* = 0 remains unstable (see the dashed line in figure 3*c*) whenever *β* > *β*_*u*_ (the invasion threshold). On the other hand, I* = 0 is globally stable (see the dotted line in figure 3*c*) for *β* < *β*_*s*_ (the persistence threshold). It is conspicuous from the figure that the transition of I* can be either continuous (*β*_*s*_ = *β*_*u*_) or discontinuous (*β*_*s*_ < *β*_*u*_) with a bistable solution.

A simplicial complex environment of interaction can again result in a discontinuous transition to the endemic state [87]. In particular, here two different facets of contagion have been encountered, at the initial stage governed by the dyadic interactions, whereas the later stage is controlled by the higher-order interactions. Theoretical analysis is provided in the homogeneous mixing limit along with rigorous computation in order to explain the associated bistable regime. By now, we all are aware of the fact that, in the case of contagions over standard network models built upon dyadic interactions, hubs play quite crucial roles. However, in higher-order networks, not only the individuals but also the groups play decisive roles. So, with a view to exploring the roles of sets of groups on the hypergraph contagion dynamics, considering heterogeneity in both hyperdegree and hyperlink cardinality, in St-Onge *et al*. [88] the authors construct a framework based upon approximate master equations analysing contagion dynamics on top of random higher-order networks. Assuming the rate of infection as a nonlinear function of the number of infectious individuals in groups, it is shown how influential groups can govern the initial dynamics as well as the final stationary state of the contagion. A mathematical formulation has been provided to analyse the linear stability of general dynamical processes on arbitrary hypernetworks on the basis of a weighted-graph projection of the hypernetwork [89]. In particular, the processes of social contagion and diffusion dynamics are dealt with. Apart from these, the study in Li *et al*. [90] investigated two competing SIS epidemic dynamics on a higher-order networked system composed of 1- and 2-simplices. Rigorous computations and the analysis of mean-field equations depicted a repertoire of dynamical features owing to the higher-order interaction. The absolute dominance of the epidemics for weak triadic infection strength and the alternative dominance for higher triadic infection strength depending on the initial seed of infection are observed.

### 4.2. Consensus formation

Consensus dynamics on higher-order networked systems (three-body systems, mainly) is analysed analytically and numerically [91], in which it was disclosed that the dynamical consequences of multibody interactions can be effective only when the interaction function is nonlinear. As a result of bringing in a nonlinear function herein, the emerged dynamics causes shifts off the state of the average system, depending on the underlying network and the initial configuration. Consensus dynamics in higher-order networks of any order is further studied in Sahasrabuddhe *et al*. [92] while contemplating a number of social processes such as homophily and peer pressure for modelling the interactions. Apart from the hypergraph models like block hypergraphs, analysis has been performed on real-world networks as well. In [93], the authors formulated a hypergraph bounded confidence model and showed the appearance of a scenario named ‘opinion jumping’, in which individuals’ opinions can jump from one cluster of opinions to another, which one does not observe in dyadic connectivity. Moreover, echo chambers are witnessed to emerge on hypergraphs with community structure. Large hyperlinks are found to be playing more decisive roles for the consensus than the small hyperlinks. Besides the computational demonstrations, the scenarios are treated mathematically. Consensus dynamics over higher-order networked systems can be investigated through the concept of *generalized Hodge Laplacians* for the instances in which the weights for lower- and higher-order interactions between simplices are different [94]. Using the Hodge decomposition, convergence can be analysed and thereafter with the techniques of algebraic topology the role of simplicial complex homology can be studied. In fact, lower- and higher-order interactions can be balanced to optimize consensus dynamics.

In the above, we have already discussed how temporal higher-order interaction patterns modulate the discrete dynamics of social contagion [82]. Let us now elaborate how temporality in network connectivity affects the continuous dynamics of consensus process developing in higher-order networks [95].

The nodal dynamics is described by the following set of equations:

4.1 $$\begin{array}{rl} {\dot{x}}_{i} & =\sum_{\;j,k}A_{ijk}\exp(l|x_{k}-x_{j}|)[(x_{j}-x_{i})+(x_{k}-x_{i})],\\ & \;i=1,2,\ldots ,N, \end{array}$$

where $A_{ijk}\in R^{N\times N\times N}$ is the adjacency tensor representing the interaction structure of the 3-hypernetwork and the term exp (*l*|*x*_*k*_ − *x*_*j*_|) is the scaling function that regulates the impacts of the *j*th and *k*th nodes on the *i*th node. Then the temporal network model of 3-regular hypergraphs is constructed by defining a sequence of adjacency tensors *A*^[1]^, *A*^[2]^, … representing the network structures at different times with *τ* being the length of the time periods between any two successive adjacency tensors.

Let us then assume a network set-up with two (individually globally connected) clusters (say, clusters ‘A’ and ‘B’) of the same size (*N* = 10 nodes) in which both intra-cluster and inter-cluster hyperlink connections exist with the nodes in cluster A (B) having the initial state *x*_A_(0) = 1 (*x*_B_(0) = 0). Further, the clusters are connected via 20 randomly placed hyper 3-links in such a way that the *p* ∈ [0, 1]-fractions of 3-links are oriented towards cluster A (i.e. the lesser number of hyperlink nodes are part of cluster A rather than part of cluster B) and the rest of the 3-links are oriented towards B. Then three different schemes are studied, namely the first-mover A, first-mover B and the aggregated scenario. To be precise, in the case of first-mover A (first-mover B), firstly for a certain time all the A-majority (B-majority) subgroups interact, then all the B-majority (A-majority) subgroups and then the entire hypernetwork interacts. For the aggregated scenario, the hypernetwork remains static and all the interactions take place concurrently.

**Figure 4.**
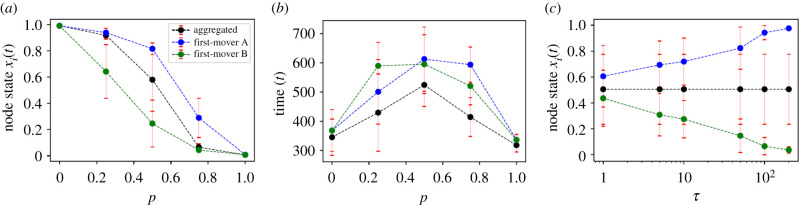
(*a*) Consensus value with respect to the parameter *p* corresponding to the scenarios of first-mover A, first-mover B and the aggregated dynamics, in which the error bars reflect one standard deviation. (*b*) Time taken to reach consensus as a function of the fraction *p*, which shows a faster rate of convergence for an asymmetric configuration. (*c*) Time scale *τ* dependence of the consensus value for *p* = 0.5. Decreasing *τ* helps in making the convergence to the aggregated dynamics. Adapted from Neuhäuser *et al*. [95].

Figure 4*a* portrays the value of consensus in terms of the node state *x*_*i*_(*t*) as a function of the fraction *p*, averaged over 10 simulations. The consensus evolving in the hypernetwork tends towards the initial opinion in cluster A or B for the aggregated scenario. Whenever *p* = 0 (*p* = 1), the connecting 3-links are oriented towards cluster B (A), making the initial opinion of cluster A (B) prevail. This outcome turns out to be qualitatively the same in the first mover cases. As far as the convergence speed is concerned, convergence is effectively faster for the asymmetric initial opinion and when orientation of the 3-links and the first-mover group line up (figure 4*b*). Further, figure 4*c* demonstrates the consensus dynamics with respect to the time scale *τ* for *p* = 0.5. It is clear from the figure that the system is more prone to converge to the aggregated dynamics whenever *τ* is small.

In Horstmeyer & Kuehn [96], the authors propose and study an adaptive voter model under higher-order interactions, specifically on a simplicial complex, that incorporates the influence of the important social factor of peer pressure. The rewiring rule of linking to agreeing nodes is adopted while focusing mainly on the 2-simplex framework. Peer pressure speeds up the transitions to both single-opinion and two-opinion states. Also, this higher-order model can exhibit multiple time scales in which the 2-simplices vanish before the active links are exhausted. In a recent work [97], a generalized dynamical model on a simplicial complex of several consensus and synchronization processes is proposed and analysed. Many behaviours are detected here for consensus dynamics that occur for dyadic interactions and also the emergence of multistability in the steady states due to this model is put forward.

### 4.3. Evolutionary game dynamics

Cooperation [98–104] is the process in which individuals function together in groups for mutual benefits; it is observed in diverse real systems including microorganisms and human society. Significant attempts have been made previously in order to explore the evolutionary game dynamics in populations subject to group interactions (see [103] and references therein). In evolutionary game theory, the higher-order interactions differ from pairwise interactions in the derivation of pay-offs. If one’s pay-off in a higher-order interaction, to some degree, is equivalent to the sum of pay-offs in interactions with each individual opponent, both higher-order and pairwise interactions essentially are the same. Otherwise, if one’s pay-off in this neighbourhood is nonlinear to the sum of pay-offs in all pairwise interactions, higher-order interactions lead to different dynamical processes. An approach to capture the higher-order interaction is a general multiplayer game, where one player’s pay-off is a function of his and all neighbours’ strategies ([105–111] furnish nice strict analytical results on multiplayer games). When the pay-off function is nonlinear to the number of cooperative neighbours, it presents the higher-order effects.

In particular, the review by Perc *et al.* [103] clarifies how larger group sizes can help in preserving cooperation in networks formed upon dyadic interactions which are often insufficient to explain all the essence of group interactions. Keeping this in mind, Burgio *et al.* [112] came up with their work on diverse hypernetworks in pursuance of having a clearer perception of the development of cooperation in networked groups while examining the evolution of cooperation in the public goods game (PGG), and demonstrated that group interactions can, indeed, enhance cooperation. The method adopted to generate the hypernetworks preserves the dyadic projection and the authors, in particular, deal with hypernetworks formed from the Holme–Kim and the Dorogovtsev–Mendes models. Besides mean-field approximation for homogeneous interactions, invasion analysis is presented for heterogeneous structures explaining how increasing the order of connections can cause higher reciprocity. The developed reciprocity is specifically due to the adopted mechanism that replaces some first-order 3-cliques with second-order triangles. The article also discusses how cooperative and non-cooperative states can coexist subject to the modality of interaction structures.

The evolutionary dynamics of the PGG is also investigated in social networks built upon higher-order interactions [113]. The study reveals that this game on uniform hypernetworks in which there is no hyperdegree–hyperdegree correlations is consistent with the replicator dynamics in the well-mixed regime. The article further incorporates heterogeneity in both order and hyperdegree, and demonstrates how these characteristic features affect the evolutionary game dynamics. As this higher-order network framework is capable of appropriately describing the group structures, the study actually has been able to depict how synergy factors depending on the group size result in critical scaling in the defection to cooperation transition. Hierarchical hypernetworks are observed to impede cooperation in a structured population. The network set-up is further employed in collaboration datasets as well. Higher-order group (three-player with two-player) interactions along with adaptation are taken into account in order to propose the adaptive simplicial snow-drift game [114]. Adaptation in the network topology and the state of the system is assumed; this explores for both mathematical and numerical treatments that, even under the higher-order structural framework, the stability of the equilibrium points remains unaltered. An evolutionary model of group choice dilemmas is proposed and analysed on hypernetworks where the decisions between a safe alternative and a risky one are taken in different sized groups; this model is capable of explaining how opinion diffuses following an imitation process [115]. Further, an organized study of a different form of strategic interaction of signalling games in populations subject to higher-order structures, namely the dynamical evolution of honesty in the sender–receiver game, was presented recently [116]. Unlike the instance of the sole presence of dyadic interactions, honesty has been witnessed to be existent even under the temptation to lie. Also, moral strategy persists even if lies favour the receiver at a cost to the sender. The evolutionary dynamics is investigated in populations based upon the assumption of a well-mixed setting, in hyper-ring as well as in real-world hypernetworks.

A different approach was adopted very recently to model evolutionary game dynamics for higher-order interactions among individuals, where, apart from the strategies of a focal player and one of the neighbours, strategies of other neighbour(s) coming out of indirect interactions also influence the game dynamics [117]. Diverse social dilemmas with different Nash equilibria being played over 1- and 2-simplices are investigated, demonstrating that such a simplicial framework results in the appearance of the non-dominant strategies and its coexistence with the dominant strategies. Further, transition from the dominant defection state to the state of cooperation with respect to the higher-order structure is established.

The two-strategy (cooperation (*C*) and defection (*D*)) two-player game configuration can be described by the following pay-off matrix:

4.2 $$\begin{array}{ccc} & C & D\\ C & R & S\\ D & T & P \end{array}$$

Each player receives a pay-off *R* = 1 (reward) under mutual cooperation and *P* = 0 (punishment) for mutual defection on the agreement of the strategies. Instead, if the players’ strategies disagree, the cooperator receives a pay-off *S* ∈ [−1, 1] (sucker), whereas the defector receives *T* ∈ [0, 2] (temptation). Concerning the network formulation, initially starting with a fully connected sub-network of *n*_0_(=5) nodes, in the next time step *m*(=1) new nodes are added. These new nodes are linked to the endpoints of randomly chosen *m* links, and thus *m* new triangles are created in the sub-network. Reiterating this step of addition of nodes, the final network of *N* nodes is constructed. The network thus formed exhibits a power-law degree distribution with an exponential generalized-degree distribution [28]. A fraction *ρ* ∈ [0, 1] of random triangles in the network are chosen to characterize actual three-body (2-simplex) interactions whereas the remaining fraction (1 − *ρ*) of triangles represents three two-body (1-simplex) interactions. A strategy matrix $\tilde{S}=\{s_{ij}\}$ is also defined that takes different values based on whether *i*th and *j*th nodes cooperate, defect or do not interact. The accumulated pay-off $\Pi_{i}$ of the *i*th node is then calculated as $\Pi_{i}=(1/k_{i})\sum_{\;j\in N_{i}}\Pi_{i,(ij)}$, where *N*_*i*_, ~*k*_*i*_ and $\Pi_{i,(ij)}$ are respectively the neighbourhood, degree and the total pay-off obtained along the link (*i*, *j*) of the *i*th node. Further, $\Pi_{i,(ij)}=(1/k_{ij})\sum_{\tau \in \Delta }\Pi_{i,(ij),\tau }$, where the set Δ comprises the *k*_*ij*_ triangles constituted by the link (*i*, *j*). Now, if *τ* is simply a sum of three 1-simplices, then $\Pi_{i,(ij),\tau }$ is obtained from game 1, the pay-off values of which are *S* = *S*_1_, *T* = *T*_1_ with *R* = 1, *P* = 0. On the other hand, if *τ* characterizes a 2-simplex, then assuming the other node to be the *k*th node that completes this simplex, $\Pi_{i,(ij),\tau }$ will be calculated from game 2 (*S* = *S*_2_, *T* = *T*_2_ with *R* = 1, *P* = 0) if *s*_*ki*_ = *s*_*kj*_. Similarly, the pay-off will be obtained from game 3 (*S* = *S*_3_, *T* = *T*_3_ with *R* = 1, *P* = 0) if *s*_*ki*_ ≠ *s*_*kj*_. This way each *i*th node obtains its pay-off $\Pi_{i}$ and subsequently updates (synchronously with others) its strategy with probability $\mathrm{Pr}=1/(1+e^{\left[(\Pi_{i}-\Pi_{\tilde{\;j}})/K\right]})$, in which $\Pi_{\tilde{\;j}}$ is the accumulated pay-off of the $\tilde{\;j}$th node.

**Figure 5.**
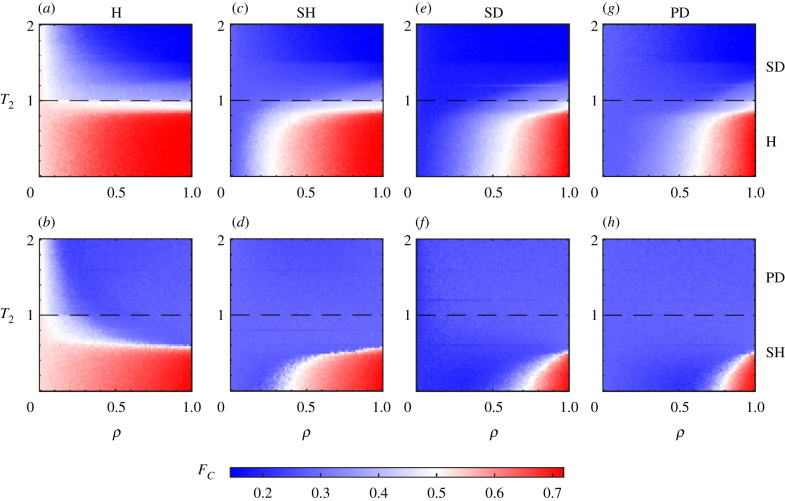
*ρ* − *T*_2_ phase diagrams depicting the frequency *F*_*C*_ of cooperators, for fixed values of *S*_2_ = 0.5 for the first row and *S*_2_ = −0.5 for the second row, with (*a*,*b*) *T*_1_ = *T*_3_ = 0.8, *S*_1_ = *S*_3_ = 0.2 that correspond to the Harmony game, (*c*,*d*) *T*_1_ = *T*_3_ = 0.8, *S*_1_ = *S*_3_ = −0.2 that represent the Stag Hunt game, (*e*,*f*) *T*_1_ = *T*_3_ = 1.2, *S*_1_ = *S*_3_ = 0.2 that define the Snowdrift game, and (*g*,*h*) *T*_1_ = *T*_3_ = 1.2, *S*_1_ = *S*_3_ = −0.2 associated with the Prisoner's Dilemma game. In the first row game 2 is SD for *T*_2_ ≥ 1 and an H dilemma when *T*_2_ ≤ 1, while in the second row game 2 is PD if *T*_2_ ≥ 1 and an SH dilemma for *T*_2_ ≤ 1. Adapted from [117].

The frequency of cooperation in the *ρ* − *T*_2_ parameter plane is shown in figure 5 while considering game 1 and game 3 to be the same (i.e. with the same *S* and *T* values). Games 1,3 are identified, respectively, by the Harmony (H), Stag Hunt (SH), Snowdrift (SD) and Prisoner’s Dilemma (PD) games from the left to the right columns of the figure. Moreover, for the upper (lower) row *S*_2_ = 0.5 (*S*_2_ = −0.5) is assumed so that game 2 represents the H (SH) dilemma whenever *T*_2_ ≤ 1 and the SD (PD) game for *T*_2_ ≥ 1. For the left-most Harmony dilemma (figure 5*a*,*b*), mutual cooperation being the Nash equilibrium, for small values of *ρ* cooperation is favoured irrespective of the specifics of game 2. With increasing *ρ*, the number of three-body interactions increases and the game 2 dynamics starts to matter, which can be any of the considered four game dynamics. For *T*_2_ ≤ 1, game 2 is either H or SH, which supports cooperation and hence *ρ* values do not matter much. However, whenever *T*_2_ ≥ 1, game 2 is SD or PD and hence the fraction of cooperators decreases. Interesting results start appearing when game 1 and game 3 correspond to SH, SD and PD (figure 5*c*–*h*). In the absence of simplicial interactions (i.e. *ρ* = 0), defection is the dominant strategy but a transition to cooperation takes place with increasing *ρ* (implying increasing higher-order interactions) whenever *T*_2_ < 1 (i.e. game 2 is either H or SH). More than the transition scenario for game 2 playing the Harmony game (for which CC is the Nash equilibrium), the transition to cooperation (even when game 1 and game 3 represent PD) for the instance of game 2 playing the SH game is noteworthy as CC and DD are the two pure Nash equilibria for the SH game.

## 5. Random walk and diffusion

With the aim of exploring the dynamics of random walks on networks beyond pairwise interactions, a family of random walks on top of simplicial complexes is defined by a Markov chain [118]. A relationship between the chain’s stationary distribution and the harmonics of the Hodge Laplacian is further established. In this context, from the higher-order homology groups and the role of orientation of the simplices to the concept of neighbours of the higher-order simplices are discussed in detail. Also in Parzanchevski & Rosenthal [119], the authors have gone through the concept of random walks on simplicial complexes. Diffusive processes in the form of a family of random walks on heterogeneous higher-order networks (hypernetworks) is brought forward while giving an analytical treatment with a general proposition for the stationary distribution of the walk [120]. A comparison analysis of this distribution with that corresponding to the traditional random walk over the associated projected network is also provided. Both model and real-world hypernetworks are treated in order to explore the proposed random walk dynamics. Specifically, from the applications in node ranking and centrality measure to classification tasks are explained. The process of diffusion on simplicial complexes is studied in Schaub *et al*. [121], who propose a normalized Hodge Laplacian matrix and demonstrate how it is associated with random walk dynamics on simplicial complexes, specifically on edges. The approach is further utilized in developing embeddings of edge flows and trajectory data and also the generalization of personalized PageRank for edges.

A class of random walks on hypernetworks is defined in such a way that the random walk process shows propensity towards hyperlinks of high or low size based upon the variation of a single size bias parameter [122]. The resulting dynamics is, in fact, capable of describing diverse hypernetwork projections on networks for different values of this bias parameter. These projections can further vary depending on this parameter and this dissimilation is examined via its effect on community structure while developing the formulation of Markov stability on hypernetworks. Let us assume a hypernetwork *H*(*V*, *E*) with *V* = {*V*_1_, *V*_2_, …, *V*_*N*_} and *E* = {*E*_1_, *E*_2_, …, *E*_*M*_} being the sets of *N* nodes and *M* hyperlinks, respectively. The incidence matrix associated with the hypernetwork is the following:

$$e_{i\alpha }=\left\{ \begin{array}{cc} 1,\; & \text{if}\;V_{i}\in E_{\alpha },\;\\ 0,\; & \text{otherwise}.\; \end{array}\right.$$

The *M* × *M* hyperlink matrix is defined as **B** = **e**^*t*^**e** in which **e**^*t*^ is the transpose of **e** and the elements *B*_*αβ*_ account for the number of nodes in $E_{\alpha }\cap E_{\beta }$. The agents are then placed on the nodes that hop at discrete times, and the weighted adjacency matrix is described as ${K_{ij}}^{\left[\sigma \right]}=\sum_{\alpha }{\left(B_{\alpha \alpha }-1\right)}^{\sigma }e_{i\alpha }e_{\;j\alpha },\;\sigma \in R,\;\forall \;i\neq j$ and ${K_{ii}}^{\left[\sigma \right]}=0$, from which the transition probabilities are computed as ${T_{ij}}^{\left[\sigma \right]}={K_{ij}}^{\left[\sigma \right]}/(\sum_{m\neq i}{K_{im}}^{\left[\sigma \right]}),\;\forall \;i\neq j$ and ${T_{ii}}^{\left[\sigma \right]}=0$. This implies how the hyperlinks of large (small) size govern the random walk dynamics for large (negative) values of the size bias parameter *σ*. A continuous random walk on top of the hypernetwork is then delineated as

5.1 $${\dot{p}}_{i}=\sum_{\;j}p_{\;j}{T_{\;ji}}^{\left[\sigma \right]}-\sum_{\;j}p_{i}{T_{ij}}^{\left[\sigma \right]},\;i=1,2,\ldots ,N,$$

in which *p*_*i*_ (*p*_*i*_(*t*)) is the probability of the agent being on the *i*th node at time *t*.

A generalization of the formulation of Markov stability [123] is further adopted in order to find the communities in the hypernetwork, by assuming a partition of the nodes into *c* non-overlapping communities, captured by the indicator matrix **C**_*N*×*c*_ where *C*_*ij*_ takes up the value 1 when the *i*th node belongs to the *j*th community, and 0 otherwise. The Markov stability *r*(*t*; **C**) then measures the goodness of **C** as a function of the time horizon of the random walk (see Carletti *et al*. [122] for detailed definitions of Markov stability).

**Figure 6.**
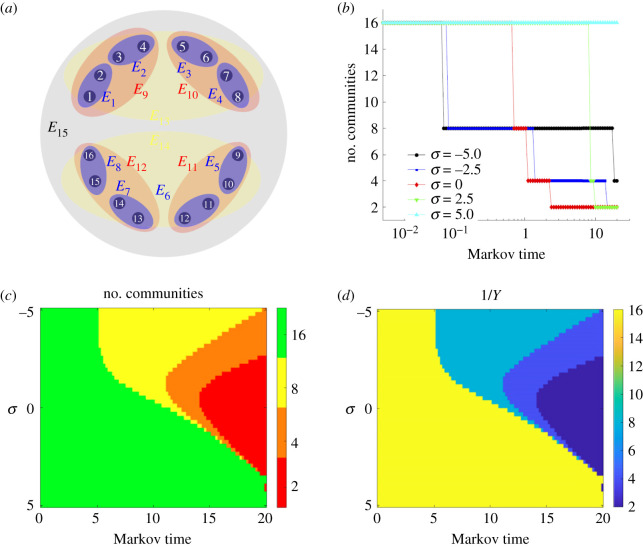
(*a*) The hypernetwork composed of 16 nodes and 15 hyperlinks, specifically there exist eight hyperlinks of size 2 (blue), four hyperlinks of size 4 (red), two hyperlinks of size 4 (yellow) and one large hyperlink of size 16 (grey). (*b*) The number of communities as a function of the Markov time for different values of the size bias parameter *σ*. (*c*) Number of communities for simultaneous variations of Markov time and *σ*. (*d*) 1/*Y* with respect to Markov time and *σ*. Adapted from Carletti *et al*. [122].

We then consider a typical hierarchical hypernetwork model comprising 16 nodes and 15 hyperlinks (figure 6*a*), the projection of which is a complete network with 16 nodes. As can be seen, there exist eight hyperlinks each of size 2, four hyperlinks containing four nodes, two hyperlinks with eight nodes and lastly the hyperlink containing all 16 nodes. In order to find the communities, Markov stability is optimized with respect to the Markov time *t* for different values of *σ* in figure 6*b*. The plots demonstrate the hierarchical structure efficiently while determining all the communities of decreasing size as the Markov time increases. Then having a look at the entries of **K**^[*σ*]^ one can calculate $\lim_{\sigma \to +\infty }{T_{ij}}^{\left[\sigma \right]}=1/15,\;\forall i,j\in \{1,2,\ldots ,16\}$, with $\lim_{\sigma \to -\infty }{T_{12}}^{\left[\sigma \right]}=1$ and $\lim_{\sigma \to -\infty }{T_{1j}}^{\left[\sigma \right]}=0$ for all other *j*. The other values follow from the symmetry in the structure of the hypernetwork. Figure 6*c* depicts the number of communities as a function of Markov time and *σ*. The algorithm yields the partition of 16 communities whenever *σ* is positive and large, and the two communities of size 8 for smaller positive *σ*. The intermediate communities are realized mainly for the negative values of *σ*, and the algorithm finally identifies the communities of size 2. Furthermore, the Simpson diversity index *Y* is computed in order to get the size of the communities, where $Y=\sum_{i=1}^{Q}{S_{i}}^{2}/N^{2}$, in which *S*_*i*_ is the number of nodes present in the *i*th group. *Y* varies from 1 (when all the nodes are in a single group) to 1/*N* (if there exist *Q* = *N* groups, each comprising a single node), whereas *Y* ∼ 1/*Q* whenever the nodes are uniformly distributed among the *Q* groups. In figure 6*d*, the value of 1/*Y* is presented for simultaneous variations in Markov time and *σ*. It is discernible that 1/*Y* = 2 is associated with the *Q* = 2 communities of size 8 as here *Y* = 2 × (8/16)^2^ = 1/2. Similarly, 1/*Y* = 4 corresponds to the *Q* = 4 communities of size 4, and 1/*Y* = 8 represents *Q* = 8 communities of size 2.

The spectral properties of a higher-order Laplacian associated with the simplicial complex model known as ‘network geometry with flavour’ (as mentioned above) are studied in Torres & Bianconi [124]. It is demonstrated that these higher-order up- and down-Laplacians can have a finite spectral dimension that depends on the order of the Laplacian. Moreover, this higher-order structure affects the diffusion dynamics taking place on this, with the spectral dimensions having meaningful influence on the return-time probability of the concerned diffusion process. Furthermore, the relation between the geometry of a network and diffusion dynamics is unravelled [125] based on the investigation of two families of models, namely NGF and ‘short-range triadic closure (STC)’. Thus far, many generalizations of different random walk models for higher-order interactions have been put forward, as discussed above. In order to explore which combination of model and network representation is best for resolving different research issues associated with diverse hypernetwork data, Eriksson *et al*. [126] derive unipartite, bipartite and multilayer network representations of hypernetwork flows with identical node-visit rates for the same random walk model. The information-theoretic and flow-based community detection algorithm Infomap is used to investigate how different hypernetwork models and network representations alter the number, size and overlap of the detected communities.

## 6. Summary and future prospects

The variants of interactions in networked systems essentially regulate the dynamical processes taking place on them. It has been demonstrated in many ways that, from synchronization to spreading dynamics, the complex interaction structure strongly decides the destiny of the concerned complex systems. However, the existing literature predominantly has dealt with pairwise networked systems, even though the underlying processes are better represented on top of higher-order structures. Only in recent times have the structural and dynamical properties of higher-order networks become a rapidly developing research field owing to their potential efficacy in describing numerous complex instances from social processes to neuroscience. In this review article, we have furnished a review of recent research endeavours that study various dynamical processes on networks beyond dyadic interactions. Our investigation clarifies how diverse the impact on different phenomena can be while higher-order connections are taken into account. The fundamental concepts of higher-order networks are briefly discussed in §2. In §3, we started by explaining how the phenomenon of synchronization gets affected by the presence of higher-order connections in the system. Social processes staring from contagion dynamics, consensus formation to evolution of cooperation are examined in §4.1, §4.2 and §4.3, respectively. The influence of higher-order interactions on random walk and diffusion dynamics is studied in §5.

Even though a number of significant developments have been made in view of analysing the role of higher-order interactions on dynamical processes, we would still like to bring forward some of the noteworthy routes of further research. For instance, there is enough variation to contribute to the understanding of temporal higher-order networks. From its structural intricacies to the analysis of different dynamics on time-varying higher-order structures is highly worth of attention. The same applies to the interdependent network frameworks, specifically the multilayer/multiplex structures along with higher-order interactions, inspection of which should be envisaged as a promising research direction. Although there exist important attempts concerning synchronization in networks beyond pairwise connectivity, the detailed analysis of cluster synchrony is missing. The specific aspect of the chimera state is mostly untouched so far, whereas these patterns have a high resemblance to several neuronal developments [127]. So, the study of chimera states in simplicial networks would be an excellent candidate for future research. Also, the study of collective behaviours of swarmalator systems with higher-order interactions could be quite interesting. Moreover, the dynamical scenarios arising from the increased complexity due to adaptivity [128] in higher-order systems require much more attentive study.

## Data Availability

This article does not contain any additional data.
